# Screening diagnostic markers of osteoporosis based on ferroptosis of osteoblast and osteoclast

**DOI:** 10.18632/aging.204945

**Published:** 2023-09-28

**Authors:** Zhihai Cao, Yuan Xue, Jiaqian Wang

**Affiliations:** 1 Department of Emergency, The Third Affiliated Hospital of Soochow University, Changzhou 213000, China; 2 Department of Orthopaedic, Zhongshan Hospital, Fudan University, Shanghai 200032, China; 3 Department of Orthopaedic, Wuxi Ninth People’s Hospital of Soochow University, Wuxi 214000, China

**Keywords:** osteoporosis, osteoblast, osteoclast, ferroptosis

## Abstract

Background: Osteoporosis is a negative balance of bone metabolism caused by the lower bone formation of osteoblasts than the bone absorption of osteoclasts. Ferroptosis plays an important role in osteoporosis, but its effects on osteoblasts and osteoclasts are still unclear.

Methods: First, we compared the osteogenic differentiation potential of MSCs and osteoclast differentiation potential of monocytes between osteoporosis mice and control. Then, we obtained gene expression profiles of MSCs and monocytes, and screened differentially expressed genes for enrichment analysis. Next, we cluster the patients with osteoporosis according to genes related to osteogenesis inhibition and osteoclast promotion. Finally, according to the expression of different subtypes of ferroptosis genes, diagnostic markers were screened and verified.

Results: The osteogenic differentiation ability of MSCs in osteoporosis mice was decreased, while the osteoclast differentiation ability of monocytes was enhanced. The DEGs of MSCs are enriched in iron ion, oxygen binding and cytokine activity, while the DEGs of monocytes are enriched in iron ion transmembrane transport and ferroptosis. Compared with the osteogenic inhibition subtype, the osteoclast promoting subtype has a higher correlation with ferroptosis, and its functions are enriched in fatty acids, reactive oxygen species metabolism and oxidoreductase activity of metal ions. SLC40A1 may be the hub gene of ferroptosis in osteoporosis by promoting osteoclast differentiation.

Conclusion: Ferroptosis may inhibit bone formation and promote bone absorption through oxidative stress, thus leading to osteoporosis. The study of ferroptosis on osteoblasts and osteoclasts provides a new idea for the diagnosis and treatment of osteoporosis.

## INTRODUCTION

Osteoporosis is a systemic osteopathy characterized by increased bone fragility and fracture prone due to low bone mass and destruction of bone microstructure [1]. About 50% of elderly women over 65 years old suffer from osteoporosis. Osteoporotic fracture brings disability, pressure sore, lung disease and other related complications, which has caused enormous pressure to society and economy [2]. The etiology and pathogenesis of osteoporosis have not been fully clarified. At present, it is believed that the bone formation of osteoblasts is less than the bone absorption of osteoclasts, resulting in a negative balance of bone metabolism, leading to osteoporosis [3].

Osteoblasts are mainly derived from bone marrow mesenchymal stem cells (MSCs). MSCs are considered as multipotent stem cells with the ability to differentiate into osteoblasts, chondrocytes and adipocytes [4]. Especially, as the source of osteoblasts, it is regulated by many factors [5]. Aging, as one of the most important factors, reduces the number and differentiation ability of MSCs, resulting in the weakening of bone formation [6]. Osteoclast is another important component, which mainly comes from monocyte-macrophage system [7]. Under the stimulation of transcription factors, cytokines and other signal factors, they fuse into multinucleated cells and finally activate into osteoclasts [8]. Hormone changes caused by aging are the cause of abnormal activation of osteoclasts [9]. Activated osteoclasts degrade bone matrix by secreting acidic substances and a variety of lysosomal enzymes [10]. In a word, the imbalance of osteoblast and osteoclast functions is still the focus of osteoporosis research.

Ferroptosis is a new type of programmed cell death which is iron dependent and different from apoptosis, necrosis and autophagy [11]. Under the action of divalent iron and ester oxygenase, it catalyzes the lipid peroxidation of unsaturated fatty acids highly expressed on the cell membrane, thus inducing cell death [12]. Ferroptosis is regulated by a variety of meticulous pathways and is associated with most degenerative diseases [13, 14]. Many studies have shown that iron ptosis plays an important role in osteoporosis, and iron overload and lipid peroxidation accumulation jointly mediate the destruction of bone homeostasis [15]. However, the mechanism of ferroptosis in osteoporosis and its effect on osteoblasts and osteoclasts are still unknown. Therefore, to explore the role of ferroptosis in the occurrence and development of osteoporosis is expected to become a new direction of anti-osteoporosis treatment.

Based on the above reasons, we first verified the weakening of osteogenic capacity and the enhancement of osteoclast capacity of osteoporosis mice *in vitro*. Subsequently, we obtained gene expression profile data in MSCs and monocytes of patients with osteoporosis. The differential expressed genes between the two kinds of cells in patients with osteoporosis and normal people were compared, and their function and pathway enrichment were analyzed. Next, we classified osteoporosis patients according to the osteogenesis inhibition gene and osteoclast promotion gene. The differential expression of ferroptosis related genes among two subtypes was discussed. Finally, the hub genes of ferroptosis in each subtype were screened and verified by experiments. The study of osteoblasts and osteoclasts based on the mechanism of ferroptosis provides a new idea and method for osteoporosis.

## MATERIALS AND METHODS

### Cell isolation and culture

All animal experimental protocols were approved by the Wuxi Ninth People’s Hospital of Soochow University. As previously mentioned, bone marrow mesenchymal stem cells (MSCs) were extracted from the femurs of normal C57/BL6 mice and 6-week-old female mice for ovariectomy, and the femurs were collected 8 weeks after the surgery [16]. Cells were collected by centrifugation and resuspended in Dulbecco modified Eagle’s medium (DMEM) - low glucose (Sigma-Aldrich, USA) containing 10% fetal bovine serum (FBS, Gibco, USA). After 3 days, the non-adherent cells were removed by washing with PBS 2–3 times. The cultures were maintained at 37°C with 5% CO_2_, and the medium was replaced 1–2 times every week. The cells after 3 passages were used in the experiment.

Similarly, bone marrow cells were extracted from the femur of normal C57/BL6 mice and ovariectomized mice. Cells were cultured overnight, then non-adherent cells were harvested, and cultured for 72 hours in medium containing 10 ng/mL macrophage colony stimulating factor (Peprotech, USA) to obtain bone marrow-derived macrophages (BMMs).

### Osteogenic differentiation and ALP staining and activity assay

To induce osteogenic differentiation, 5 × 10^4^ cells were inoculated into each well of the 12 well plate. Added 50 μg/mL ascorbic acid and 10 mM of β-glycerol phosphate medium was used as osteogenic medium and was set as OB group.

After 7 days of osteogenesis induction, osteoblasts were stained with BCIP/NBT ALP Colour Development Kit (Beyotime Biotech, China) according to the manufacturer’s plan. The activity of ALP was measured by the absorbance of ALP Assay Kit (Nanjing Jiancheng Bioengineering Institute, China).

### Osteoclast differentiation and TRAP staining

To induce osteoclast differentiation, 1 × 10^4^ BMMs were inoculated into each well of 96 well plate. Add 30 ng/mL M-CSF and 50 ng/mL RANKL (R&D Systems) as osteoclast culture medium, and set it as RANKL group.

After 5 days of culture, the osteoclasts were stained with TRAP according to the instructions of TRAP Kit (Sigma-Aldrich, USA). The images of TRAP-positive cells with nuclei ≧ 3 were obtained using a light microscope (Zeiss, Germany) and the area of TRAP-positive osteoclasts were counted in six randomly chosen fields of view.

### Verification of osteogenesis and osteoclast related genes by qRT-PCR

After the cells were induced and cultured according to the above, the total RNA of the cells was extracted and reverse transcribed according to the instructions of TRIzol Kit (Beyotime, China). Universal SYBR Green Supermix (Bio-Rad, USA) was used for quantitative gene analysis. The relative gene expressions were calculated by the 2^−ΔΔCt^ method. The gene primer sequences of osteogenic markers ALP, RUNX2 and OCN, osteoclast markers TRAP and CTSK are listed in Supplementary Table 1 [17].

### Datasets collection and preprocessing

We searched the Gene Expression Omnibus (GEO) database (https://www.ncbi.nlm.nih.gov/geo/) with the keyword “osteoporosis”. Inclusion criteria are as follows: (1) Gene expression profile of homo sapiens; (2) Osteoporosis with complete data. Finally, we screened three datasets for further study. GSE35959 includes 5 normal samples and 5 osteoporosis samples from MSCs. GSE100609 contains 4 normal samples and 4 osteoporosis samples from monocytes. GSE152073 contains 44 whole blood tissue samples from patients with osteoporosis.

### Screening and enrichment analysis of differential expressed genes

Differential expression genes (DEGs) between osteoporosis patients and normal people were identified by “limma” package. *P*-value < 0.05 and |log2FC| > 1 was considered statistically significant. Volcanic and heat maps visualize the results and show the difference level of DEGs.

The Gene Ontology (GO) and Kyoto Encyclopedia of Genes and Genomes (KEGG) were used to analyze the functions and pathways of DEGs. This process was completed by the “clusterProfiler” package. Five different biological processes, cell components, molecular functions and KEGG pathways were screened by *p*-value < 0.05.

### Identification of ferroptosis related genes and construction of protein–protein interaction (PPI) network

405 ferroptosis related genes were downloaded from the FerrDb database, and the ferroptosis related DEGs of osteoblasts and osteoclasts were extracted through the intersection of Venn diagrams. The online tool STRING was used to analyze the PPI network of ferroptosis related DEGs. Cytoscape software (v3.8.0) makes a better visualization of PPI network, and extracts hub subnetworks using the cytoHubba plug-in.

### Cluster analysis of different subtypes

In order to further study the role of ferroptosis in different osteoporosis patients, we conducted cluster analysis on the whole blood dataset. We first obtained the osteogenesis inhibiting genes and osteoclast promoting genes from the MSigDB database. According to the expression of osteogenesis inhibiting genes and osteoclast promoting genes in different samples, “ConsenseClusterPlus” based on the “pam” method was used to cluster the samples. Finally, all tissue samples were divided into C1 subtype with decreased bone formation and C2 subtype with increased bone resorption on the basis of empirical cumulative distribution function diagram and tSNE diagram.

### Enrichment analysis and gene expression difference of two subtypes

Gene set enrichment analysis (GSEA) was used to compare the differential expression of two different subtypes of osteoporosis, and four significantly different GO biological processes, cellular components, molecular functions and KEGG pathways were screened with *p*-value < 0.05. The differences in the expression of osteoblast inhibiting genes and osteoclast promoting genes of the two subtypes, as well as the differences in the expression of genes related to ferroptosis, are all shown by violin diagrams.

### Evaluation of immune cells infiltration

CIBERSORT algorithm was used to evaluate the infiltration of immune cells in the whole blood of osteoporosis patients, and the score of immune infiltration cells in each sample was calculated according to the expression profile of the dataset. Violin diagram visualized the immune cells between the two subtypes. Pearson method was used to analyze the correlation of different immune cells, and the correlation heat map was used for visualization.

### Verification of ferroptosis related genes by qRT-PCR

The differential genes in the two subtypes were used as diagnostic markers for ROC analysis. The area under the curve (AUC) >0.75 indicates that the marker has good diagnostic value.

In order to further verify the validity of diagnostic markers for osteoporosis, we performed qRT-PCR analysis on ferroptosis genes. MSCs and monocytes of osteoporosis mice were obtained according to the above method and tested. All experiments were performed independently in triplicate. Supplementary Table 2 lists the primer sequences of ferroptosis related genes.

### Statistical analysis

All R packages are implemented through RStudio (v4.1.3). All data are shown as means (standard error of the mean (SEM)), and statistical analysis was performed by independent-samples *t*-tests on GraphPad Prism 9 (GraphPad Software, USA).

### Availability of data and material

The data used to support the findings of this study are included within the article.

## RESULTS

### Osteogenic differentiation of MSCs in osteoporosis mice

**Figure 1:**
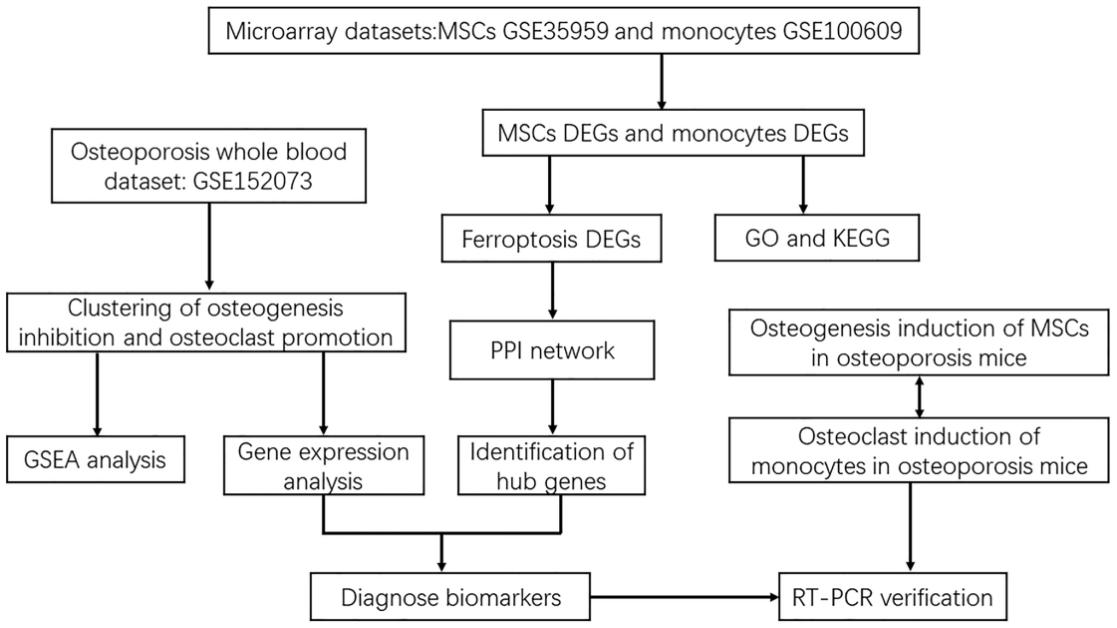
Flowchart of screening diagnostic markers of ferroptosis in osteoporosis based on osteoblasts and osteoclasts.

**Figure 2:**
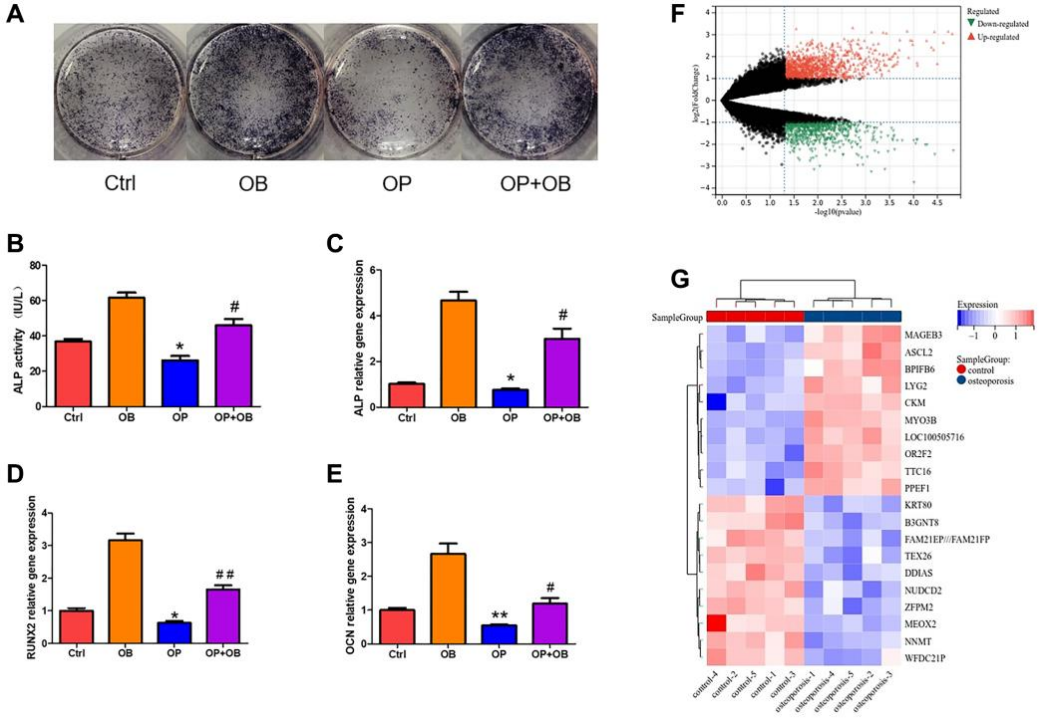
**The difference of MSCs between osteoporosis and control.** (**A**) ALP staining of osteogenic differentiation of MSCs from osteoporosis mice and control mice. (**B**) Quantitative analysis of ALP activity. (**C**–**E**) The gene expression of ALP, RUNX2 and OCN was detected by qRT-PCR after MSCs induced osteogenesis. (**F**) Volcanic map shows DEGs of MSCs in patients with osteoporosis and control. (**G**) Heat map shows the top twenty up-regulated and down-regulated DEGs. (Compared with Ctrl group, the statistically significant difference was ^*^p < 0.05, ^**^p < 0.01, and compared with OB group, it was ^#^*p* < 0.05, ^##^*p* < 0.01, *n* = 3).

The research flowchart of this study was shown in Figure 1. After 7 days of osteogenesis induction, compared with normal MSCs, there were fewer ALP positive cells in OP group, with a statistically significant difference (Figure 2A). In OP group, the ability of MSCs to differentiate into osteoblasts decreased, corresponding to a lower ALP activity value (Figure 2B). The mRNA expression levels of ALP, RUNX2 and OCN in OP group were also significantly lower than those in control group (Figure 2C–2E).

### Screening and enrichment analysis of DEGs in MSCs

In MSCs of normal group and osteoporosis group, 1067 up-regulated DEGs and 732 down-regulated DEGs were found (Figure 2F). The heat map shows 10 up-regulated genes and 10 down-regulated genes with the most obvious differences (Figure 2G).

**Figure 3:**
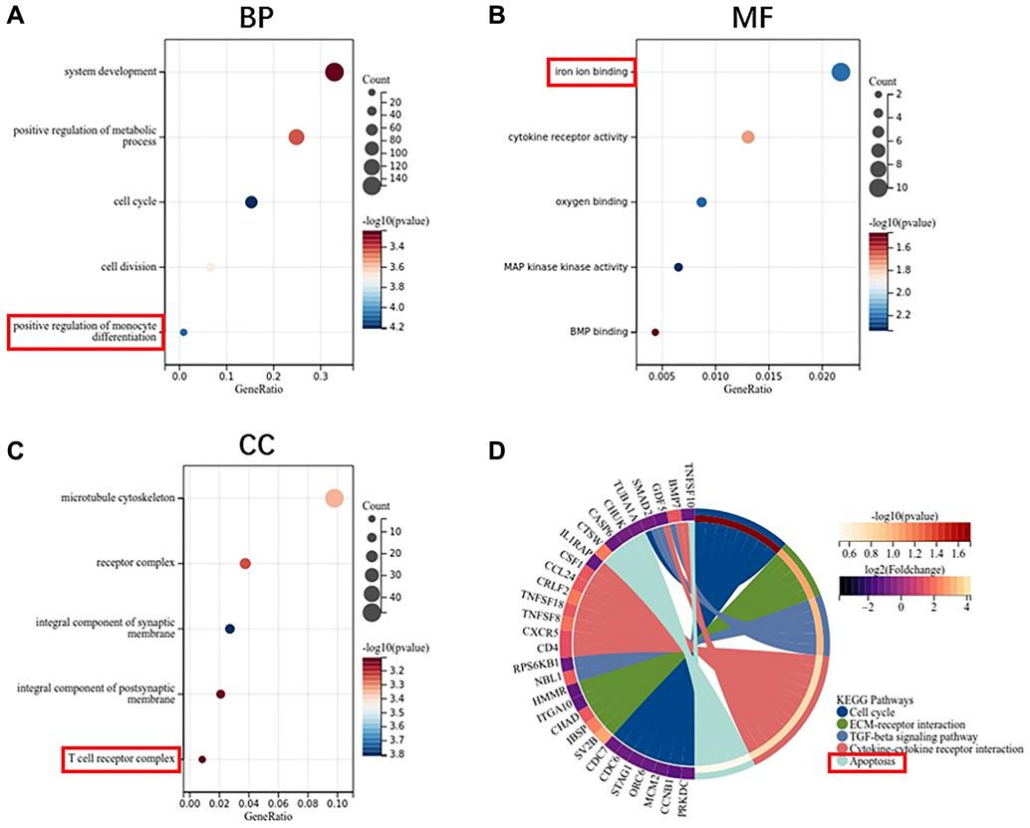
**Function enrichment analysis of DEGs in MSCs.** (**A**) Biological process of DEGs. (**B**) Molecular function of DEGs. (**C**) Cellular component of DEGs. (**D**) KEGG pathway of DEGs.

GO enrichment analysis showed that the biological process of DEGs mainly focused on system development, metabolic process and cell cycle (Figure 3A). Molecular functions are concentrated in iron ion binding, cytokine receptor activity and oxygen binding (Figure 3B). Cell components are concentrated in microtubule cytoskeleton, receptor complex and synaptic membrane (Figure 3C). KEGG pathway analysis showed that ECM-receptor interaction, TGF- beta signaling pathway, cytokine receptor interaction and apoptosis are the main enrichment pathways (Figure 3D).

### Osteoclast differentiation of monocytes in osteoporosis mice

**Figure 4:**
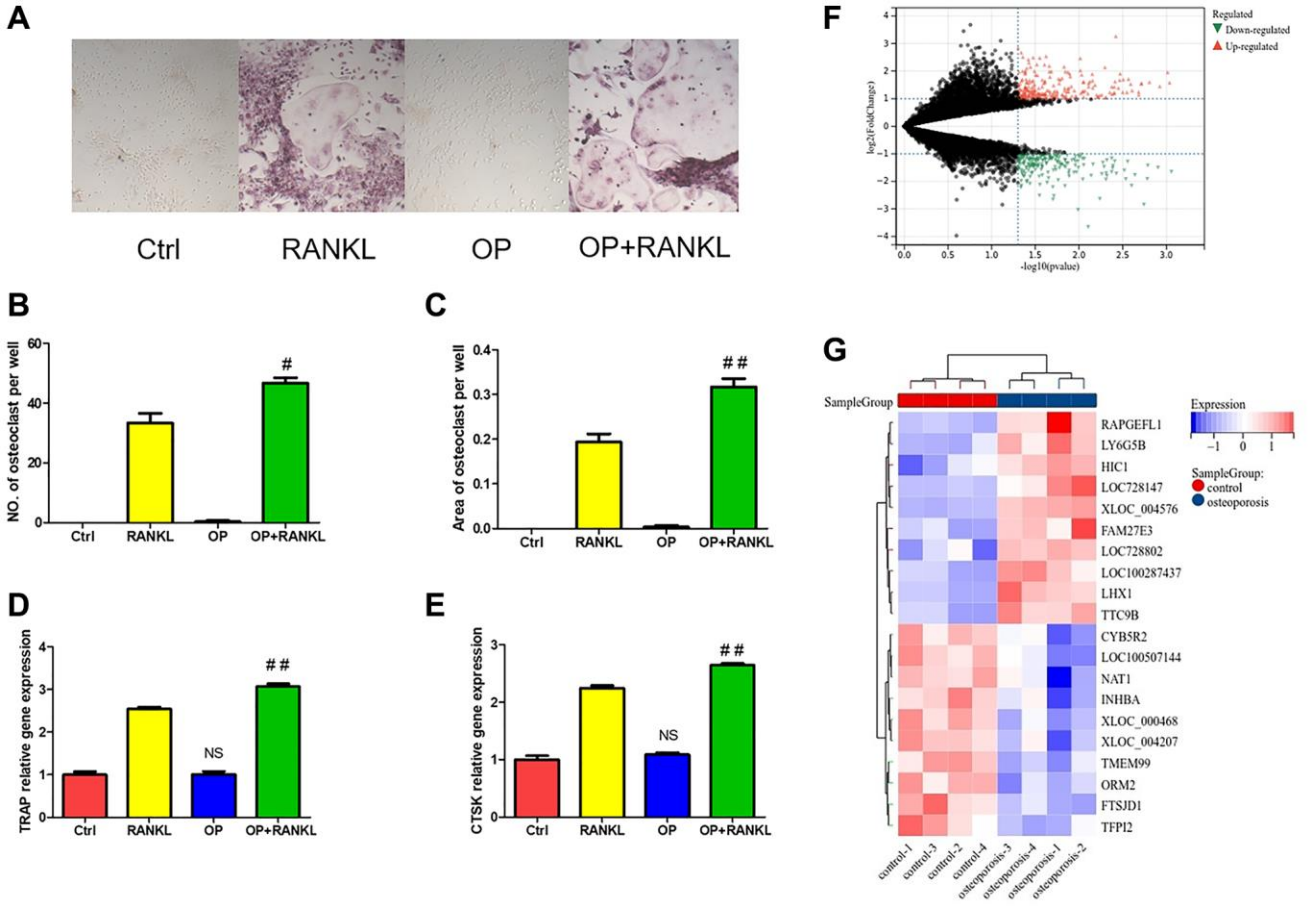
**The difference of monocytes between osteoporosis and control.** (**A**) TRAP staining of osteoclast differentiation of monocytes from osteoporosis mice and control mice. (**B**, **C**) Quantitative analysis of TRAP positive osteoclasts. (**D**, **E**) The gene expression of TRAP and CTSK was detected by qRT-PCR after monocytes induced osteoclast. (**F**) Volcanic map shows DEGs of monocytes in patients with osteoporosis and control. (**G**) Heat map shows the top twenty up-regulated and down-regulated DEGs. (NS means no significance compared with the Ctrl group, and compared with RANKL group, the statistically significant difference was ^#^*p* < 0.05, ^##^*p* < 0.01, *n* = 3).

After 5 days of osteoclast induction, compared with the control group, there were more TRAP positive cells in OP group, with a statistically significant difference (Figure 4A). Quantitative results showed that the number of osteoclasts were more and the area of osteoclasts were larger in OP group (Figure 4B, 4C). The mRNA expression levels of TRAP and CTSK in OP group were also significantly higher than those in control group, indicating that the osteoclast differentiation ability of OP group was enhanced (Figure 4D, 4E).

### Screening and enrichment analysis of DEGs in monocytes

Similarly, 232 up-regulated and 195 down-regulated DEGs were identified in monocytes of normal and osteoporosis groups (Figure 4F). Figure 4G shows the heat map of the ten most different up-regulated and down-regulated genes.

**Figure 5:**
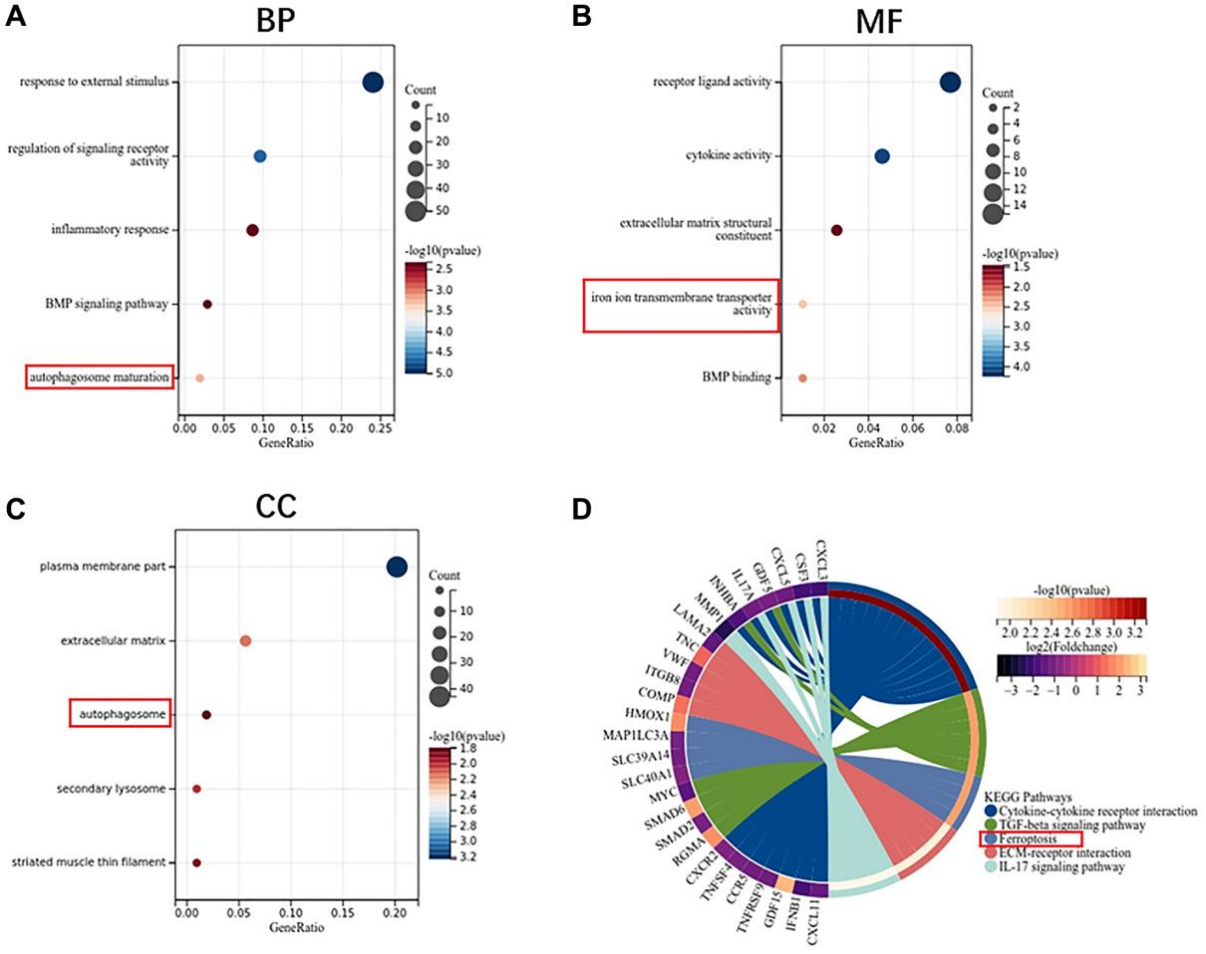
**Function enrichment analysis of DEGs in monocytes.** (**A**) Biological process of DEGs. (**B**) Molecular function of DEGs. (**C**) Cellular component of DEGs. (**D**) KEGG pathway of DEGs.

GO enrichment analysis shows that the biological process of monocyte DEGs mainly focuses on the response to external stimulus and inflammatory response (Figure 5A). Molecular functions are concentrated in receptor ligand activity, cytokine activity and iron ion transmembrane transporter activity (Figure 5B). Cell components are concentrated in the plasma membrane part, extracellular matrix and autophagosome (Figure 5C). KEGG pathway analysis showed that cytokine receptor interaction, TGF-beta signaling pathway, ferroptosis and ECM-receptor interaction are the main enrichment pathways (Figure 5D).

### Identification of ferroptosis related genes and construction of PPI network

**Figure 6:**
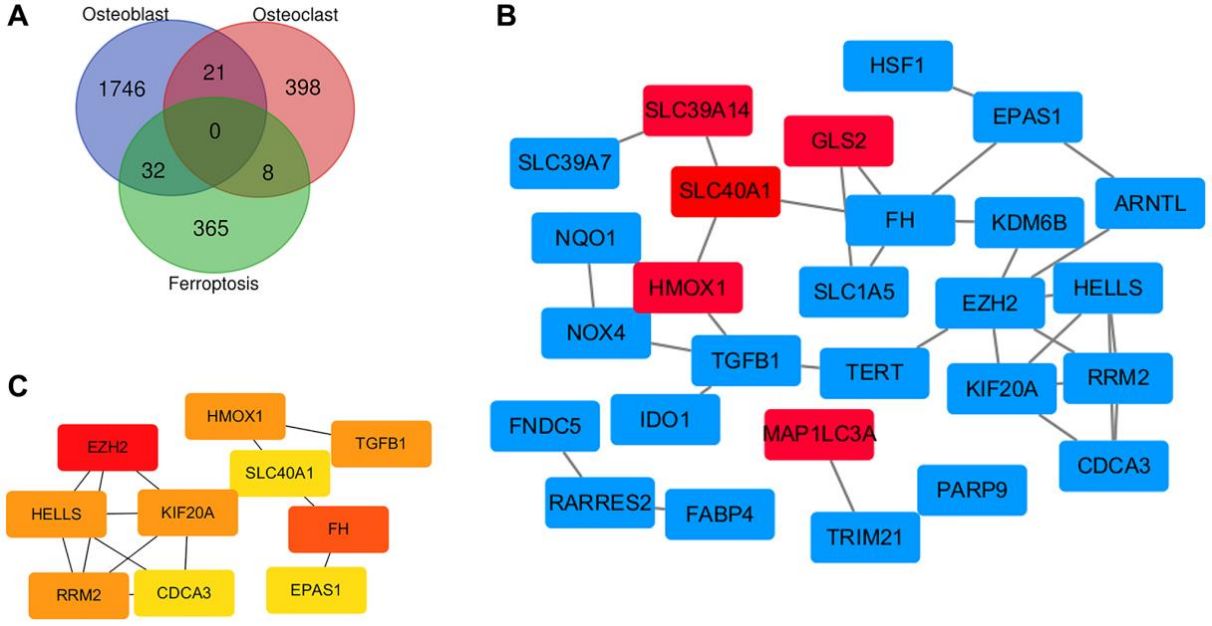
**Identification of ferroptosis related genes and construction of PPI network.** (**A**) Identification of ferroptosis related genes in osteoporosis by Venn diagram. (**B**) Cytoscape construct PPI network of ferroptosis related genes in osteoporosis. (**C**) Screening hub genes through the PPI network.

As shown in the Venn diagram, there are 32 overlapping genes between ferroptosis related genes and the DEGs of MSCs, and 8 overlapping genes between ferroptosis related genes and the DEGs of monocytes (Figure 6A). Through the visualization of the Cytoscape software, we constructed an interaction network between ferroptosis DEGs coding proteins, which is composed of 27 nodes and 34 edges. Among them, 22 DEGs of MSCs are marked red, and 5 DEGs of monocytes are marked blue (Figure 6B). We used CytoHubba to identify the hub genes. According to the degree, MCC and MNC, we identified the top 10 nodes (Figure 6C).

### Identification of subtypes of osteogenesis inhibition and osteoclast promotion in osteoporosis

**Figure 7:**
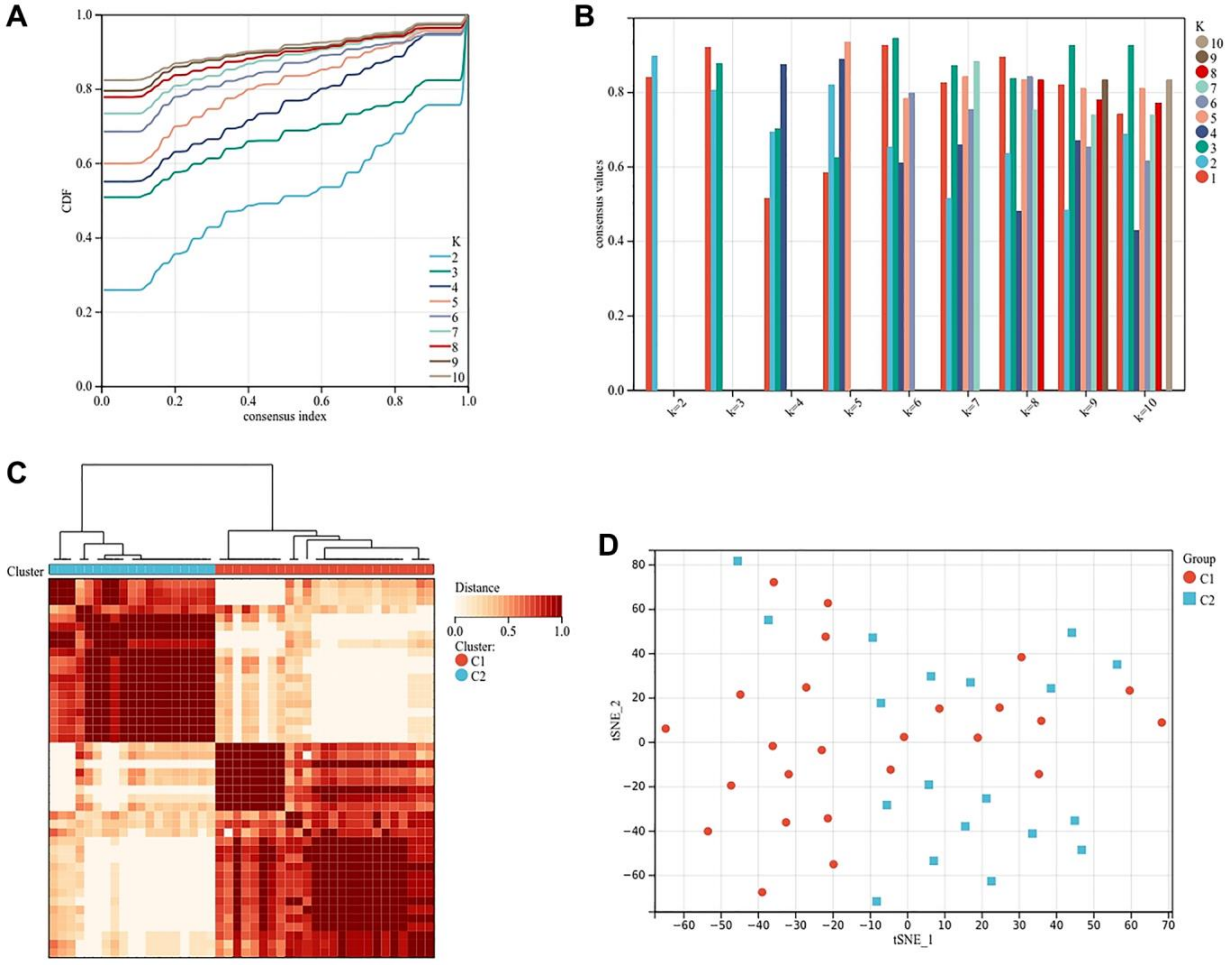
**Cluster analysis based on the expression of genes related to osteogenesis inhibition and osteoclast promotion.** (**A**) Clustering cumulative distribution function (CDF) curve. (**B**) Samples clustering consistency, determine k = 2. (**C**) Clustering Heatmap, cluster 1 (*n* = 25) and cluster 2 (*n* = 19). (**D**) tSNE diagram of two subtypes.

In order to further explore the role of ferroptosis in osteoblasts and osteoclasts, we clustered the samples according to the expression of genes related to osteogenesis inhibition and osteoclast promotion in different samples. According to the area under the cumulative distribution function curve and the average consistency evaluation within the cluster group, the consistency is better when the specific cluster number k = 2 (Figure 7A, 7B). Forty-four osteoporosis samples were divided into two subtypes: cluster 1 (*n* = 25) and cluster 2 (*n* = 19) (Figure 7C). The tSNE diagram shows that there are differences between the two subtypes, which again proves the reliability of clustering (Figure 7D).

### Enrichment analysis of two subtypes

**Figure 8:**
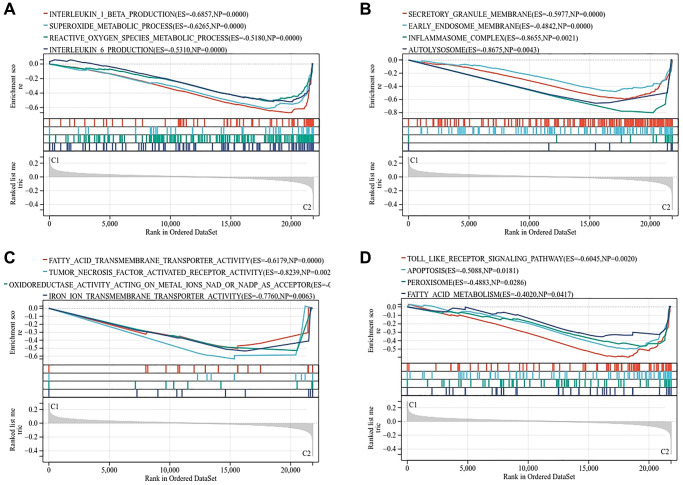
**Gene set enrichment analysis of two subtypes.** (**A**) Biological process. (**B**) Molecular function. (**C**) Cellular component. (**D**) KEGG pathway.

We analyzed the gene expression profiles of the two subtypes by GSEA. The results showed that the biological process of C2 subtype was enriched in superoxide metabolic process, reactive oxygen species metabolic process and interleukin production (Figure 8A). Cell components are enriched in inflammasome complex and autolysosome (Figure 8B). Molecular functions are enriched in fatty acid, iron ion transmembrane transporter activity and oxidoreductase activity acting on metal ions (Figure 8C). The analysis of KEGG pathway showed that apoptosis, peroxisome and fatty acid metabolism are the main enrichment pathways (Figure 8D).

### Gene expression and immune cell infiltration of two subtypes

**Figure 9:**
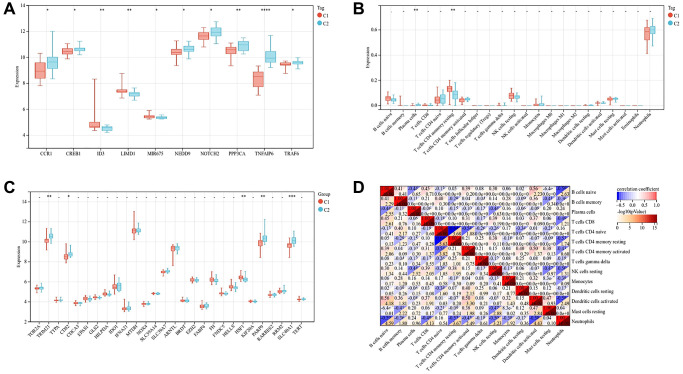
**Gene expression and immune cell infiltration of two subtypes.** (**A**) Expression of genes related to osteogenesis inhibition and osteoclast promotion in two subtypes. (**B**) Expression of genes related to ferroptosis in two subtypes. (**C**) Violin diagram shows the difference of immune cells between two subtypes of osteoporosis. (**D**) Correlation heatmap shows the relationship between immune cells. (The statistically significant difference was ^*^p < 0.05, ^**^p < 0.01, ^***^p < 0.001).

In order to further study the two subtypes, we compared the expression of genes related to osteogenesis inhibition and osteoclast promotion in the two subtypes. Osteogenesis inhibitory genes ID3, LIMD1 and MIR675 were relatively high in C1 subtype, and osteoclast promoting genes CCR1, CREB1, NEDD9, NOTCH2, PPP3CA and TRAP6 were relatively high in C2 subtype (Figure 9A). The expression of TRIM21, CD82, PARP9 and SLC40A1 genes related to ferroptosis was relatively high in C2 subtypes (Figure 9B).

Neutrophils are the main immune cells in the whole blood of patients with osteoporosis. Compared with C1 subtype, C2 subtype contained more plasma cells and fewer T cells with CD4 memory resting, *p*-value < 0.01 (Figure 9C). The correlation heat map showed that plasma cells were negatively correlated with B cells naive and NK cells resting, T cells CD4 memory resting was negatively correlated with T cells CD4 naive and neutrophils, with the correlation coefficient >0.4 (Figure 9D).

### Screening and validation of diagnostic markers

**Figure 10:**
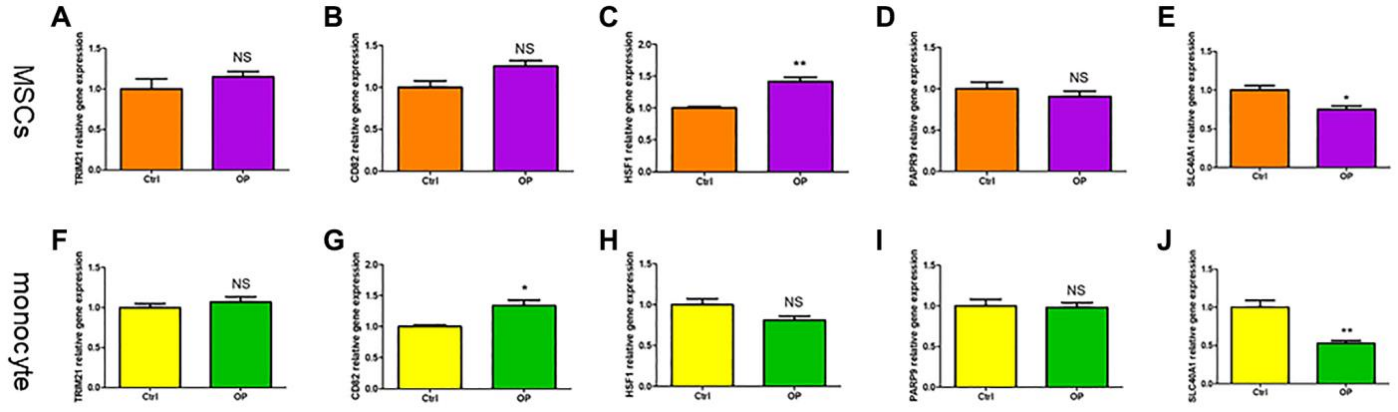
**The validity of ferroptosis diagnostic markers for osteoporosis was verified by qRT-PCR.** (**A**–**E**) Detection of ferroptosis related genes expression in MSCs of mice by qRT-PCR. (**F**–**J**) Detection of ferroptosis related genes expression in monocytes of mice by qRT-PCR. (NS means no significance, statistically significant difference was ^*^p < 0.05, ^**^p < 0.01).

TRIM21, CD82, HSF1, PARP9 and SLC40A1 are differentially expressed genes in the two subtypes. Compared with the control group, the expression of HSF1 in MSCs of osteoporosis mice was higher, and the expression of SLC40A1 was lower (Figure 10A–10E). However, in monocytes, the expression level of CD82 is relatively high in osteoporosis mice, and the expression level of SLC40A1 is relatively low (Figure 10F–10J). The AUC of SLC40A1 is 0.85, which has good diagnostic value (Supplementary Figure 1). SLC40A1 is also a hub gene in PPI network, which may be a diagnostic marker of ferroptosis in osteoporosis.

## DISCUSSION

Osteoporosis refers to the imbalance between osteoblasts and osteoclasts in the process of bone remodeling. The number and activity of osteoblasts are reduced, the number of osteoclasts is increased, and functions are active, leading to the reduction of bone formation and the increase of bone resorption [18]. In our study, we first confirmed that the osteogenic differentiation ability of MSCs in osteoporosis mice was lower than that of normal control group. Sui found that the number, expression of active molecules and osteogenic differentiation potential of MSCs in elderly patients with osteoporosis were lower than those in normal people, which was the same as our experimental results *in vitro* [19]. MSCs have osteogenic differentiation potential, but show different characteristics in the disease state. These characteristics may be related to pathological microenvironment, such as inflammation and tumor [20]. Previous studies have confirmed that osteoporosis may be an inflammatory disease, and the inflammatory microenvironment leads to the aging and functional decline of MSCs [21]. The function enrichment of DEGs in osteoporosis patients showed that iron binding, cytokine receptor activity and oxygen binding were the main molecular functions in the cell cycle and metabolism of MSCs.

In addition, we also found that the osteoclast differentiation ability of monocytes in osteoporosis mice was also higher than that in the control group. Because the estrogen level in postmenopausal women is reduced, the inhibitory effect on osteoclasts is weakened, and the number and activity of osteoclasts are significantly increased [22]. It has been found that estrogen deficiency can induce the expansion of T cells secreting TNF-α, thereby promoting MSCs to secrete more proinflammatory cytokines [23]. The up-regulation of proinflammatory cytokines, such as IL-1β, IL-6 and TNF-α further activates osteoclasts and participates in bone resorption [24]. Our research also shows that the biological process of monocytes is mainly concentrated in the response to external stimulus and inflammatory response. What attracts our attention is that the molecular function of monocytes is enriched in iron ion transmembrane transporter activity and the pathway of monocytes is enriched in ferroptosis. Enrichment analysis showed that both osteoblast precursor cells-MSCs and osteoclast precursor cells-monocytes were closely related to iron metabolism and inflammation.

Osteoporosis is closely related to abnormal iron metabolism [25]. The disorder of iron metabolism will affect the homeostasis of bone itself. Iron overload will accelerate the apoptosis of osteoblasts and the differentiation of osteoclasts, while iron deficiency will affect the metabolism of collagen and vitamin D [26]. Ferroptosis is a non-apoptotic way of cell death, which is mainly characterized by the accumulation of iron dependent reactive oxygen species (ROS) in cells, thus promoting cell death [27]. We hypothesized that ferroptosis would reduce bone formation of osteoblasts and increase bone absorption of osteoclasts. According to the expression of osteogenesis inhibiting gene and osteoclast promoting gene, we divided osteoporosis patients into C1 subtype with decreased bone formation and C2 subtype with increased bone resorption. C2 subtypes are significantly enriched in the biological process of reactive oxygen species metabolic, interleukin production and molecular functions of fatty acid, iron ion transmembrane transporter activity, oxidoreductase activity acting on metal ions. This proves once again that ferroptosis plays an important role in osteoporosis, especially in osteoclasts. It is reported that high iron level can significantly promote the production of RANKL, increase the proportion of RANKL/OPG, and finally enhance the differentiation of osteoclasts [28]. In another study, the expression of ROS, RANKL and IL-6 in osteoclasts increased, indicating that ROS produced by iron overload reaction may promote the expression of RANKL and enhance bone resorption [29]. Iron chelating agent can effectively inhibit iron aggregation, thus reducing osteoclast differentiation and preventing bone loss [30]. We also found the correlation between osteoporosis and fatty acid metabolism in functional enrichment analysis. In Supplementary Figure 2, we calculated the difference of clinical characteristics between the two subtypes. The C2 subtype is related to blood phosphorus, blood glucose and weight, but has no statistical significance. The latest research shows that free fatty acids are not the driving factor of ferroptosis, and ferroptosis is ultimately driven by the peroxidation of specific membrane lipids [31]. This also side verifies the reliability of our research.

In the past, the serum indicators of osteoporosis patients mainly relied on the osteogenic metabolic indicators P1NP and osteoclast metabolic indicators β-CTX [32]. In recent years, evaluating the changes of iron metabolism indicators in patients with osteoporosis has positive clinical value for comprehensive analysis of osteoporosis related factors [33]. Kim found that the increase of serum ferritin level was significantly related to the decrease of bone mass in different bone parts and the increase of incidence of osteoporosis and fracture, especially in women ≥45 years old [34]. Based on the correlation of iron metabolism with osteoblasts and osteoclasts, we screened diagnostic markers of ferroptosis in osteoporosis. SLC40A1 is a gene encoding the only iron transporter protein (FPN) in mammalian cells, which is related to iron accumulation *in vivo* [35]. FPN deletion increased iron levels in osteoclasts, increased osteoclast production and reduced bone mass [36]. Some research results show that SCL40A1 is related to the development of Osteoclast. The SLC40A1 knockout mice show abnormal bone phenotype, and the differentiation of Osteoclast can also be reduced by supplementing exogenous iron [37, 38]. In our PCR results, only SLC40A1 was identified as a diagnostic marker because its expression in MSCs and monocytes of osteoporosis mice was lower than that in the control. Unfortunately, we have not verified it in patients with osteoporosis, which may be our next research direction.

## CONCLUSION

Ferroptosis may inhibit bone formation and promote bone absorption through oxidative stress, thus leading to osteoporosis. SLC40A1 promotes osteoclast differentiation and is the hub gene of ferroptosis in osteoporosis.

## Supplementary Materials

Supplementary Figures

Supplementary Tables
