# Supplementary material for: Screening diagnostic markers of osteoporosis based on ferroptosis of osteoblast and osteoclast
Source: Aging (Albany NY). 2023 Sep 28;15(18):9391–407. doi: 10.18632/aging.204945 (PMC10564410; doi:10.18632/aging.204945)
Supplement: Supplementary Figures [file aging-15-204945-s001.pdf]

SUPPLEMENTARY FIGURES

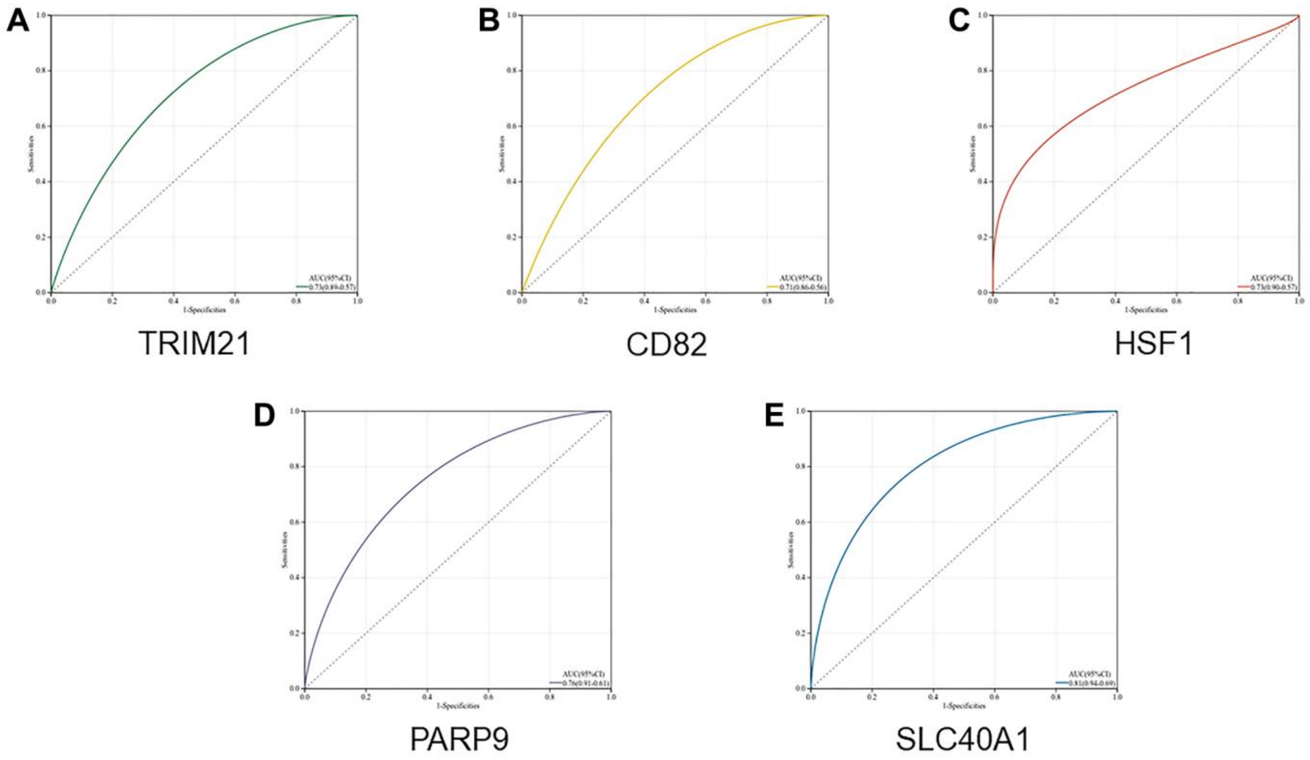

Supplementary Figure 1. Analyze the validity of diagnostic markers through ROC curve. (**A**) ROC curve of TRIM21. (**B**) ROC curve of CD82. (**C**) ROC curve of HSF1. (**D**) ROC curve of PARP9. (**E**) ROC curve of SLC40A1.

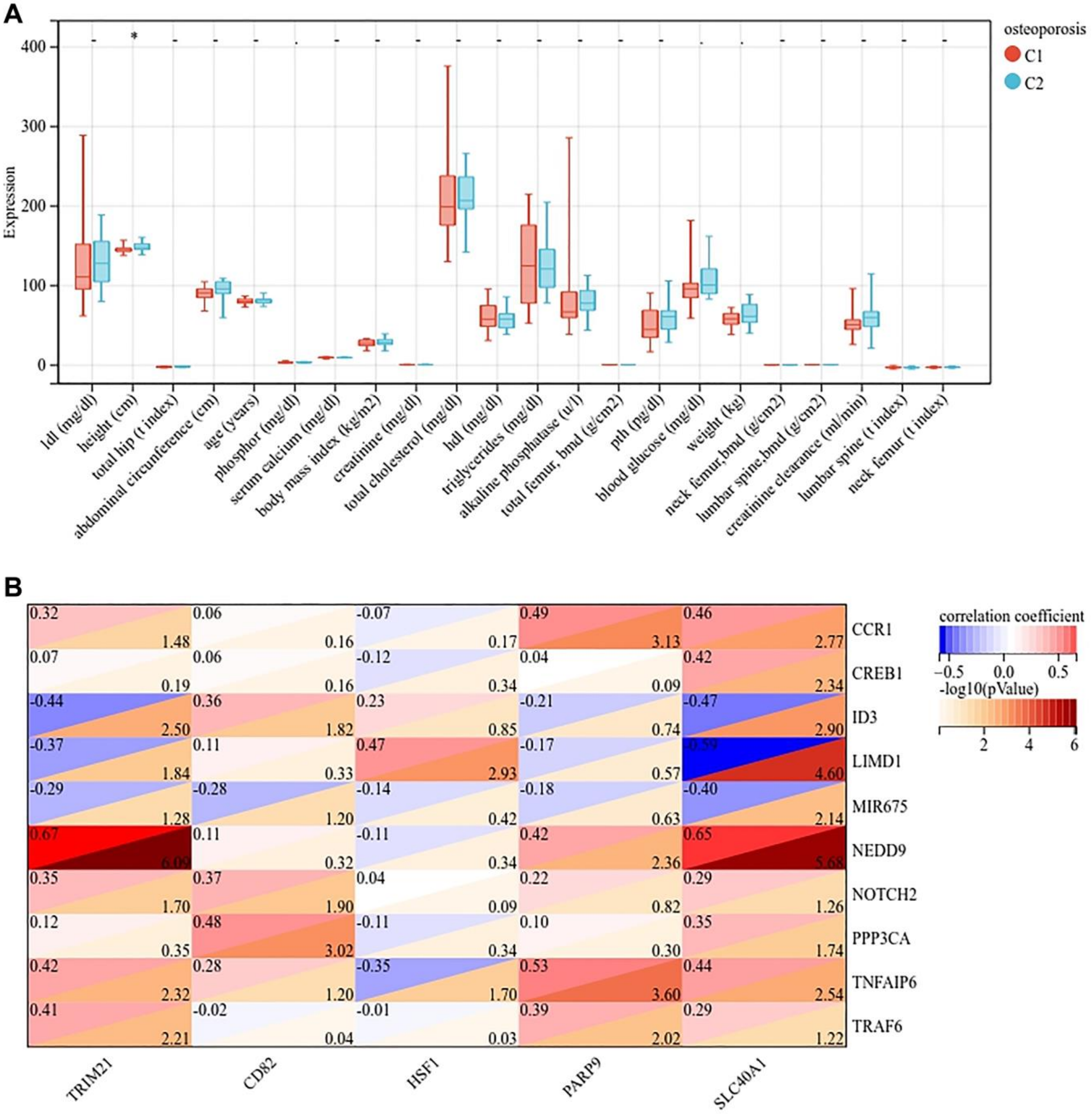

Supplementary Figure 2. (**A**) Comparison of clinical characteristics of two subtypes. (**B**) Correlation heatmap of ferroptosis genes and osteogenesis and osteoclast genes. (· means $p < 0.1$, statistically significant difference was $^{*}p < 0.05$).
