# Supplementary material for: Screening diagnostic markers of osteoporosis based on ferroptosis of osteoblast and osteoclast
Source: Aging (Albany NY). 2023 Sep 28;15(18):9391–407. doi: 10.18632/aging.204945 (PMC10564410; doi:10.18632/aging.204945)
Supplement: Supplementary Tables [file aging-15-204945-s002.pdf]

SUPPLEMENTARY TABLES

Supplementary Table 1. Primers of mice genes for qPCR.

Genes	F Sequences (5′—3′)	R Sequences (5′—3′)
GAPDH	ACCCAGAAGACTGTGGATGG	CACATTGGGGGTAGGAACAC
TRAP	CTGGAGTGCACGATGCCAGCGACA	TCCGTGCTCGGCGATGGACCAGA
CTSK	AGCAGAACGGAGGCATTGACTC	TTTAGCTGCCTTTGCCGTGGC
ALP	TGTTACTGCCAGGACCCATA	CTTCCTTGATGGTCTCCACA
RUNX2	ATAGTCCTTCCTACCCCAATTTCC	GATGAATTGGATGGTCTTGGTCC
OCN	AGTTGACGGACCCCAAAAGA	GGACAGCCCAGGTCAAAGG

Supplementary Table 2. Primers of ferroptosis genes for qPCR.

Genes	F Sequences (5′—3′)	R Sequences (5′—3′)
GAPDH	ACCCAGAAGACTGTGGATGG	CACATTGGGGGTAGGAACAC
TRIM21	AGAGAGACTTCACCTGTTCTGT	TCAGTTCCCCTAATGCCACCT
CD82	TACAAACCTCATCCAGCTCG	TCTTCACAATGAGCTGGTTG
HSF1	GACCAAGCTGTGGACCCTC	CACTTTCCGGAAGCCATACAT
PAPR9	CGGATGTCCCTGGCAGAAGAA	ACTCGACACCTTGCGATCCAA
SLC40A1	AACAAGCACCTCAGCGAGAG	CACATCCGATCTCCCCAAG
